# Supplementary material for: Inhibiting acute, axonal DLK palmitoylation is neuroprotective and avoids deleterious effects of cell-wide DLK inhibition
Source: Nat Commun. 2025 Apr 3;16:3031. doi: 10.1038/s41467-025-58036-6 (PMC11968826; doi:10.1038/s41467-025-58036-6)
Supplement: Supplementary file 1 — Supplementary Information [file 41467_2025_58036_MOESM1_ESM.pdf]

Supplementary Material for Zhang et al., “Inhibiting acute, axonal DLK palmitoylation is neuroprotective and avoids deleterious effects of cell-wide DLK inhibition”.

Supplementary Tables:

Table S1: Data for 33 compounds from primary screen that reduced DLK-GFP puncta number (puncta per NLS; P/NLS) or intensity (vesicle average intensity; VAI) in a follow-up triplicate assay. All compounds from primary screen that reduced both P/NLS and VAI below the 2SD cut-off were re-assayed in triplicate at the indicated concentrations using the HEK 293T cell assay shown in Figure 2. Data for 33 compounds that reduced (i) P/NLS by >30% and/or (ii) VAI by >10% in this follow-up assay are plotted here. None of these compounds reduced mCherry NLS count by >30% in the primary screen, although a subset of compounds did so in this follow-up screen. However, all compounds that met criteria (i) and/or (ii) were assayed in neurons (Fig 4). Significance of dose dependence was assessed by two-sided, unpaired t test of triplicate determinations at the indicated concentrations. 'YES' indicates $p<0.05$ for the indicated readout. One compound (**22**) approached but did not reach statistical significance ($p=0.053$) but was followed up based on manual inspection of images.

		Puncta/NLS (P/NLS)						Vesicle Average Intensity (VAI)						mCherry NLS					
		10uM		3uM		1uM		10uM		3uM		1uM		10uM		3uM		1uM	
Hit No	Maybridge CAT_No	AVE	%	AVE	%	AVE	%	AVE	%	AVE	%	AVE	%	AVE	%	AVE	%	AVE	%
**1**	BTB00713SC	1.0692	**30.9**	1.5149	2.1	1.8492	-19.5	412.48	**11.8**	447.16	4.4	463.59	0.9	327.56	1.8	343.78	-3.0	317.61	4.8
**2**	BTB07602SC	0.4973	**43.9**	0.8242	6.9	1.3373	-51.0	379.59	**17.3**	423.24	7.8	461.23	-0.4	569.83	-18.0	592.33	-22.6	457.5	5.3
**3**	CD01955SC	0.8855	**42.8**	1.4518	6.2	1.6234	-4.9	414.4	**11.4**	452.98	3.2	461.6	1.3	349	-4.6	347	-4.0	346.72	-3.9
**4**	CD02036SC	0.7465	**47.5**	1.3466	5.4	1.6279	-14.4	392.79	**13.0**	441.05	2.3	447.08	0.9	397.28	-30.5	358.33	-17.7	305.56	-0.3
**5**	HTS01118SC	0.3442	**52.8**	0.6201	14.9	0.5203	28.6	416.81	**10.7**	448.08	4.0	456.79	2.2	532.78	2.5	496.83	9.1	544	0.5
**6**	HTS04553SC	0.5668	22.3	0.8891	-21.9	0.8293	-13.7	416.92	**10.7**	459.94	1.5	472.36	-1.2	586.44	-7.3	500.11	8.5	547.78	-0.2
**7**	HTS05834SC	0.5778	**33.6**	1.0464	-20.2	1.1744	-34.9	384.13	**16.4**	442.33	3.7	464.85	-1.2	386.11	7.5	394.72	5.4	391.72	6.2
**8**	HTS06895SC	0.333	**36.6**	0.4224	19.6	0.4385	16.6	338.79	**13.7**	375.84	4.3	379.91	3.3	141.22	**32.2**	174.44	16.3	167.61	19.6
**9**	HTS09450SC	0.3044	**47.9**	0.4266	**27.0**	0.4336	**25.8**	357.98	**13.8**	379.69	8.6	418.85	-0.9	173.78	28.0	161.72	33.0	136	43.7
**10**	RH01693SC	0.1384	**75.5**	0.4706	16.6	0.5894	-4.4	347.97	**14.0**	386.82	4.4	391.11	3.4	195.44	**31.1**	288.61	-1.7	363.72	-28.2
**11**	RJC01887SC	0.3168	**39.7**	0.4729	10.0	0.4734	9.9	343.22	**12.6**	366.43	6.7	378.86	3.5	238.83	-14.6	277.56	-33.2	300.06	-44.0
**12**	RJC03231SC	0.2794	**51.4**	0.5167	10.2	0.5674	1.4	342.37	**15.0**	379.06	5.9	396.73	1.5	260.78	-19.8	278.11	-27.8	303.89	-39.6
**13**	RJF01488SC	0.2529	**57.4**	0.4258	**28.2**	0.5488	7.5	314.53	**24.3**	367.36	**11.6**	385.75	7.2	183.67	16.5	247.89	-12.8	298.61	-35.8
**14**	S09668SC	0.2255	**53.1**	0.3005	**37.6**	0.4236	12.0	363.3	**12.3**	382.36	7.7	413.19	0.2	108.67	**32.8**	151.28	6.5	226.56	-40.0
**15**	SEW02506SC	0.1549	**70.5**	0.4844	7.9	0.4633	11.9	295.78	**24.7**	380.43	3.1	380.92	3.0	140.17	**32.7**	290.67	-39.5	254.78	-22.3
**16**	SEW04994SC	0.3161	**45.1**	0.5019	12.8	0.5868	-2.0	339.02	**15.8**	379.61	5.7	381.64	5.2	143.17	**34.2**	237.06	-8.9	266.17	-22.3
**17**	TL00098SC	0.2466	**56.3**	0.3236	**42.7**	0.3969	**29.7**	345.8	**14.5**	359.27	**11.2**	363.72	**10.1**	239.89	15.5	241.83	14.8	240.67	15.2
**18**	BTB10119SC	0.1355	**68.9**	0.2054	**52.9**	0.2933	**32.7**	331.41	**12.9**	353.04	7.2	358.02	5.9	175.67	18.5	216.17	-0.3	274.78	-27.5
**19**	DP01302SC	0.3267	**33.5**	0.386	**21.5**	0.4841	1.5	352.67	**10.9**	364.8	7.8	380.77	3.8	247.67	-6.0	308.11	-31.9	351.06	-50.3
**20**	DP01312SC	0.2905	**40.9**	0.3713	**24.5**	0.4325	12.0	340.65	**13.9**	362.65	8.4	375.95	5.0	188.67	19.2	251.78	-7.8	303.78	-30.0
**21**	HTS03456SC	0.2746	**44.2**	0.4391	10.7	0.4216	14.3	348.89	**11.8**	380.4	3.9	380.88	3.7	190.22	18.6	263.83	-12.9	254.28	-8.8
**22**	**HTS06879SC**	0.489	**30.6**	0.5798	17.7	0.6428	8.7	340	**10.9**	362	5.4	370	3.0	339	10.0	353	6.4	401	-6.5
**23**	**HTS07222SC**	0.4651	**33.9**	0.5926	15.9	0.6183	12.2	344	9.9	354	7.3	365	4.6	307	18.5	359	4.8	419	-11.4
**24**	**JP00899SC**	0.4902	**31.9**	0.58	19.5	0.6714	6.8	350	**11.3**	365	7.6	394	0.3	353	15.5	393	5.9	417	0.1
**25**	**SPB06618SC**	0.4877	**30.1**	0.595	14.7	0.5658	18.9	370	5.5	367	6.2	357	8.8	314	25.0	390	7.0	391	6.7

Puncta/NLS (P/NLS)		Vesicle Average Intensity (VAI)		Puncta/NLS (P/NLS)		Vesicle Average Intensity (VAI)	
Two-sided t test p value (10uM vs 3uM)	Two-sided t test p value (10uM vs 1uM	Two-sided t test p value (10uM vs 3uM	Two-sided t test p value (10uM vs 3uM	Significant (p<0.05; 10uM vs 3uM)?	Significant (p<0.05; 10uM vs 1uM)?	Significant (p<0.05; 10uM vs 3uM)?	Significant (p<0.05; 10uM vs 1uM)?
0.200237042	0.015479033	0.000156106	5.42732E-05	No	Yes	Yes	Yes
0.034187334	0.068603724	0.000293754	0.000120442	Yes	No	Yes	Yes
0.017863684	0.010897145	0.002110103	0.000120425	Yes	Yes	Yes	Yes
0.008123647	0.000349897	0.000441648	0.000193304	Yes	Yes	Yes	Yes
0.006774154	0.028730697	0.027780357	0.010073624	Yes	Yes	Yes	Yes
0.020251689	0.008503687	0.000740012	0.000228499	Yes	Yes	Yes	Yes
0.101070046	3.47441E-05	0.000176546	0.00117739	No	Yes	Yes	Yes
0.025417155	0.08762099	0.004458907	0.001734084	Yes	Yes	Yes	Yes
0.013210091	0.021205699	0.145795253	0.001641274	Yes	Yes	No	Yes
0.000983675	7.00698E-06	0.040782724	0.030979643	Yes	Yes	Yes	Yes
0.001167883	0.008574219	0.018093635	0.013673253	Yes	Yes	Yes	Yes
0.000433836	0.000380744	0.040981026	0.008407421	Yes	Yes	Yes	Yes
0.121774502	0.018453967	0.015098643	0.005601917	No	Yes	Yes	Yes
0.064363993	0.003964267	0.476648547	0.106546815	No	Yes	No	No
2.94993E-05	1.40616E-05	6.13176E-05	0.000266028	Yes	Yes	Yes	Yes
0.011648544	0.000109817	0.002454764	0.001792068	Yes	Yes	Yes	Yes
0.174063145	0.000968109	0.24814161	0.12237083	No	Yes	No	No
0.002956061	0.000107026	0.269221291	0.159683296	Yes	Yes	No	No
0.083220847	0.000208166	0.478851768	0.051740421	No	Yes	No	No
0.087508006	0.002577692	0.003637383	0.000995199	No	Yes	Yes	Yes
0.01636082	0.022972922	0.066714414	0.061385498	Yes	Yes	No	No
0.077415102	0.052963803	0.147299967	0.079390477	No	No	No	No
0.013263908	0.010278061	0.28249547	0.037693241	Yes	Yes	No	Yes
0.008867482	0.004507162	0.215219606	0.010452567	Yes	Yes	No	Yes
0.011772931	0.02291348	0.774366545	0.234708626	Yes	Yes	No	No

		Puncta/NLS						Vesicle Average Intensity						mCherry NLS					
		10uM		3uM		1uM		10uM		3uM		1uM		10uM		3uM		1uM	
Hit No	Enamine CAT_No	AVE	%	AVE	%	AVE	%	AVE	%	AVE	%	AVE	%	AVE	%	AVE	%	AVE	%
**26**	**Z198022656**	0.3215	**48.7**	0.4533	**27.6**	0.4973	**20.6**	327	8.7	337	6.0	343	4.5	290	14.8	342	-0.5	350	-2.8
**27**	**Z223817830**	0.4148	**40.0**	0.637	7.8	0.6262	9.4	350.91	7.2	366.09	3.2	370.74	2.0	558.5	7.5	638.33	-5.7	658.83	-9.1
**28**	**Z31189063**	0.3657	**47.1**	0.5175	**25.1**	0.6189	10.4	336.79	**11.0**	354.13	6.4	367.72	2.8	639.89	-5.9	640.33	-6.0	665.83	-10.2
**29**	**Z228869724**	0.3182	**44.8**	0.5192	9.9	0.565	2.0	338.04	**9.2**	360.33	3.2	368.06	1.1	339.22	19.3	448.44	-6.7	438.39	-4.3
**30**	**Z387344412**	0.0914	**84.1**	0.2464	**57.3**	0.4511	**21.7**	313.55	**15.7**	329.7	**11.4**	354.44	4.8	323.5	23.0	435.39	-3.6	443.33	-5.5
**31**	**Z102923060**	0.4212	**33.8**	0.5805	8.8	0.5946	6.6	338.48	**10.6**	359.97	5.0	368.48	2.7	384.39	22.0	500.44	-1.5	539	-9.4
**32**	**Z53051092**	0.3412	**46.4**	0.4957	**22.1**	0.5952	6.5	329.24	**13.1**	344.45	**9.1**	360.45	4.8	411.28	16.6	479.67	2.7	523.56	-6.2
**33**	**Z203923506**	0.3958	**37.4**	0.5333	15.7	0.5568	12.0	332.86	**12.1**	349.71	7.7	359.35	5.1	349.94	20.5	431.61	1.9	438.61	0.3

Puncta/NLS (P/NLS)		Vesicle Average Intensity (VAI)		Puncta/NLS (P/NLS)		Vesicle Average Intensity (VAI)	
Significant (10uM vs 3uM)?	Significant (10uM vs 1uM)?	Significant (10uM vs 3uM)?	Significant (10uM vs 1uM)?	Significant (10uM vs 3uM)?	Significant (10uM vs 1uM)?	Significant (10uM vs 3uM)?	Significant (10uM vs 1uM)?
0.010084207	0.002781135	0.030477395	0.013624442	Yes	Yes	Yes	Yes
0.00075509	0.000156581	0.192061253	0.093754703	Yes	Yes	No	No
0.000287651	0.000736937	0.012096072	0.001204011	Yes	Yes	Yes	Yes
0.005969417	0.001012632	0.046824735	0.028739305	Yes	Yes	Yes	Yes
0.00510193	0.000571629	0.05171787	0.005352325	Yes	Yes	No	Yes
0.007290761	0.010847434	0.069851978	0.044790943	Yes	Yes	No	Yes
0.000334049	7.04548E-05	0.005343674	0.003104271	Yes	Yes	Yes	Yes
0.003898437	0.000724336	0.011713569	0.016052992	Yes	Yes	Yes	Yes

Supplementary Table 2. Small molecule screening data

Category	Parameter	Description
Assay	Type of assay	High content imaging
	Target	Transfected wild-type GFP-tagged DLK (wtDLK-GFP) expressed in HEK293T cells
	Primary measurement	Number of wtDLK-GFP puncta per transfected cell normalized to number of transfected cells (quantified using mCh-NLS marker), average intensity of wtDLK-GFP puncta
	Key reagents	Wt DLK-GFP cDNA, mCh-NLS cDNA, DAPI (nuclear marker), HEK293T cells.
	Assay protocol	HEK293T cells were seeded in poly-lysine coated 96 well plates (Greiner Bio-One, black walled chimney-wells), transfected and treated with 2BP (positive control ‘tool’ compound, 10 μM final concentration), library compounds (10 μM final concentration) or DMSO vehicle control at 2 h post-transfection. Maybridge or Enamine library compounds were spotted onto 96 well plates at 10 mM in DMSO and resuspended in 200 μL pre-warmed DMEM. 40 μL of diluted compound was then added to cells in 160 μL of DMEM (containing glutamax, 10% FBS and antibiotics). Cells were returned to a tissue culture incubator for a further 14 h at 37 °C. Medium was then aspirated and cells were fixed in 4% PFA (1x PBS) for 20 mins at RT, washed once with PBS and stained with 300 nM DAPI for 5 mins at RT, followed by 2 washes of PBS.<br>High Content imaging was performed using an ImageXpress micro high content imaging system (Molecular Devices, Downingtown, PA) driven by MetaXpress software. Six images per well were acquired in each of three channels (DAPI, FITC, TRITC) at 10X magnification in an unbiased fashion. Images were analyzed using the MetaXpress ‘Multiwavelength Scoring’ (for mCherry-NLS signals) and ‘Transfluor’ modules (for DLK-GFP signals). Data were exported to a spreadsheet using the AcuityXpress software package (Molecular Devices). Three metrics were used: DLK puncta (“Total Puncta Count” option, from DLK-GFP signal), DLK vesicle average intensity (VAI; the intensity of the punctate DLK-GFP signal) and total number of transfected cells (from mCherry-NLS signal). The first and last of these metrics were combined to calculate DLK-GFP Puncta per NLS (P/NLS).
	Additional comments	
Library	Library size	28,400 (20,000 compounds from Maybridge Screening Collection, 8400 from Enamine Screening Collection
	Library composition	Molecular weights < 500 (average MW = 325). All logP values < 5 (average logP value = 3.2), < 5 H-bond donors, < 10 H-bond acceptors, number of rotatable bonds < 8 (average # of rotatable bonds < 5)
	Source	Maybridge and Enamine Screening Collections, purchased by Temple University’s Moulder Center for Drug Discovery
	Additional comments	
Screen	Format	96-well plates
	Concentration(s) tested	10 μM (primary screen). 10 μM, 3 μM, 1 μM (confirmation screen)

	Plate controls	DMSO vehicle, 2BP (positive control 'tool' compound, 10 µM final concentration). 16 wells per plate were used for these controls, typically A1-D1 and E12-H12 for DMSO, and E1-H1 and A12-D12 for 2BP.
	Reagent/ compound dispensing system	Manual (8-channel automated pipettor) for plating, transfection, drug treatment and fixation steps. Manual 96-channel pipetting system (Rainin Liquidator 96) for wash steps.
	Detection instrument and software	ImageXpress micro high content imaging system (Molecular Devices, Downingtown, PA) driven by MetaXpress software
	Assay validation/QC	Initial validation (Z-factors) described in PMID: 308424714. For scaled-up conditions in this study, each of the first four screening runs also contained two additional 96-well plates transfected to express wtDLK-GFP and mCh-NLS as above. Alternating blocks of 3x4 wells in each plate were treated with either DMSO vehicle or 2BP (positive control). After fixation and analysis as above, Z-factors were calculated for the P/NLS and VAI readouts for each plate. Z-factors for main screening runs were calculated from the 8 DMSO- and 8 2BP-treated wells per plate described above.
	Correction factors	None
	Normalization	Data are plotted relative to the average of each readout for all compounds tested, after exclusion criteria described below
	Additional comments	Compounds that reduced mCh-NLS number by >30% (versus vehicle control from same plate) were excluded due to likely broad inhibition of transcription and/or translation (1723 compounds). Compounds that increased P/NLS by >1.8 relative to all controls were also excluded (33 compounds).
Post-HTS analysis	Hit criteria	Reduction of P/NLS and VAI by >2SD relative to mean of all compounds assessed
	Hit rate	1.32%
	Additional assay(s)	Hits from primary screen (375 compounds) were re-assayed in triplicate at 3 concentrations. Compounds that reduced P/NLS by >30% or VAI by >10%, and which showed dose-dependence in one or both readouts were used for orthogonal assay in primary neurons. Hit rate for this confirmation step was 0.09% (33 of 375 compounds).<br>Initial orthogonal assay in primary neurons assessed ability of compounds (10 µM) to inhibit c-Jun phosphorylation (p-c-Jun) induced by trophic factor deprivation (TD, known to require palmitoyl-DLK). Compounds that significantly inhibited TD-induced p-c-Jun were then assessed for their ability to maintain cell body viability and axon integrity after prolonged TD.
	Confirmation of hit purity and structure	Structural integrity of library members was originally confirmed by the vendors using ^{1}H-NMR and LC/MS and reconfirmed by Temple's Moulder Center using LC/MS
	Additional comments	

Table S3 Absorption, Drug Metabolism and Pharmacokinetic (ADME) data for compounds 8 and 13.

Upper Table: The indicated compounds were subjected to a MDCK-MDR1 assay (performed as in Methods) to predict their likely potential to cross the blood-brain barrier (BBB). BBB permeability for both compounds is predicted to be high.

Lower Table: The indicated compounds were assessed for their solubility in aqueous solution, their stability in liver microsomes and their inhibition of CYP450 enzymes, as described in Methods. **8** showed low-to-moderate maximum kinetic aqueous solubility, low-to-moderate stability in liver microsomes in the presence of NADPH and good stability to enzymatic hydrolysis in liver microsomes in the absence of NADPH. **8** also showed moderate inhibitory potency against CPY3A4 and CYP2D6, likely due to the presence of the isoxazole and/or thiazole moiety. **13** showed low maximum kinetic aqueous solubility and low stability in liver microsomes but did not inhibit CYP450 enzymes.

MDCK-MDR1 Assay Results

**MC #**	**Recovery %**		**A to B** Papp (x 10^{-6} cm/sec)	**B to A** Papp (x 10^{-6} cm/sec)	**Efflux Ratio** (A-B/B-A)	**Brain Penetration Classification**
	A-B	B-A				
**8**	57.5	69.9	11.5	10.6	0.93	High
**13**	39.2	64.5	18.4	17.8	0.97	High

In Vitro ADME Data

**Compound #**	**Structure**	**Max. Aq. Solubility**	**Microsomal Stability ($t_{1/2}$, minutes)**		**CYP450 Inhibition (IC_{50}, nM)**		
		uM	+ NADPH	- NADPH	3A4	2D6	2C9
**8**	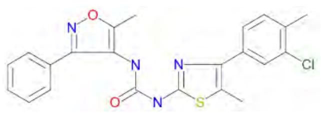	7.8	14.1	101%	1,150	6,804	> 10000
**13**	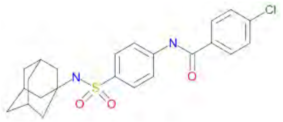	. < 2	2.2	. 2%	> 10000	> 10000	> 10000

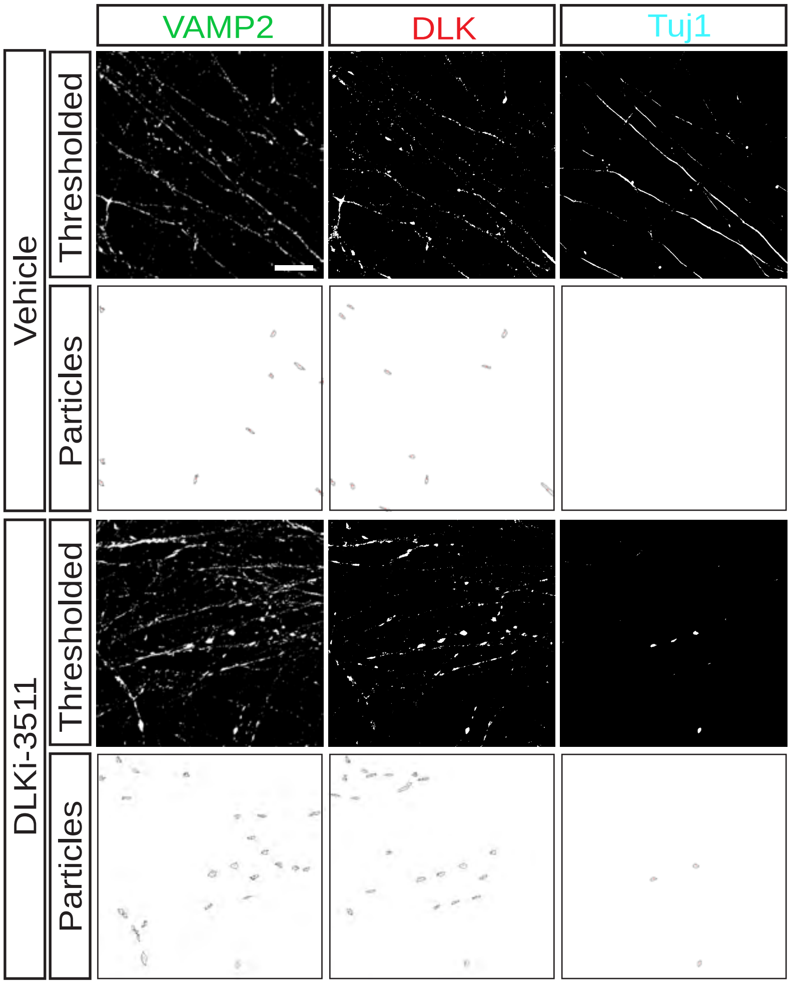

Figure S1: Example of thresholding and particle count analysis images, related to Figure 1. Images of individual channels from Figure 1B were subjected to auto-thresholding and particle count analysis, as described in Methods. Images of these image processing steps, including outlined puncta/accumulations (‘Particles’), whose area is then quantified relative to the total thresholded area for each given signal, are shown. Scale bar: 20µm, all panels. Representative images are shown. Similar results were obtained from 4 independent cultures

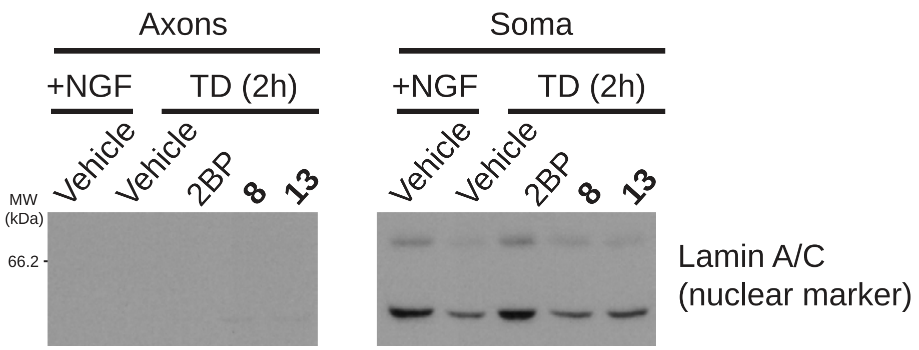

Figure S2: Confirmation of fidelity of axonal preparation for Fig 2C. Lysates of axonal fractions used in Fig 2C were subjected to SDS-PAGE and western blotting with Lamin A/C antibody (nuclear marker) side-by-side with somal fractions from the same cultures. Lamin A/C are essentially absent from axonal fractions Represtentative images are shown. Similar results were obtained from 6 independent cultures

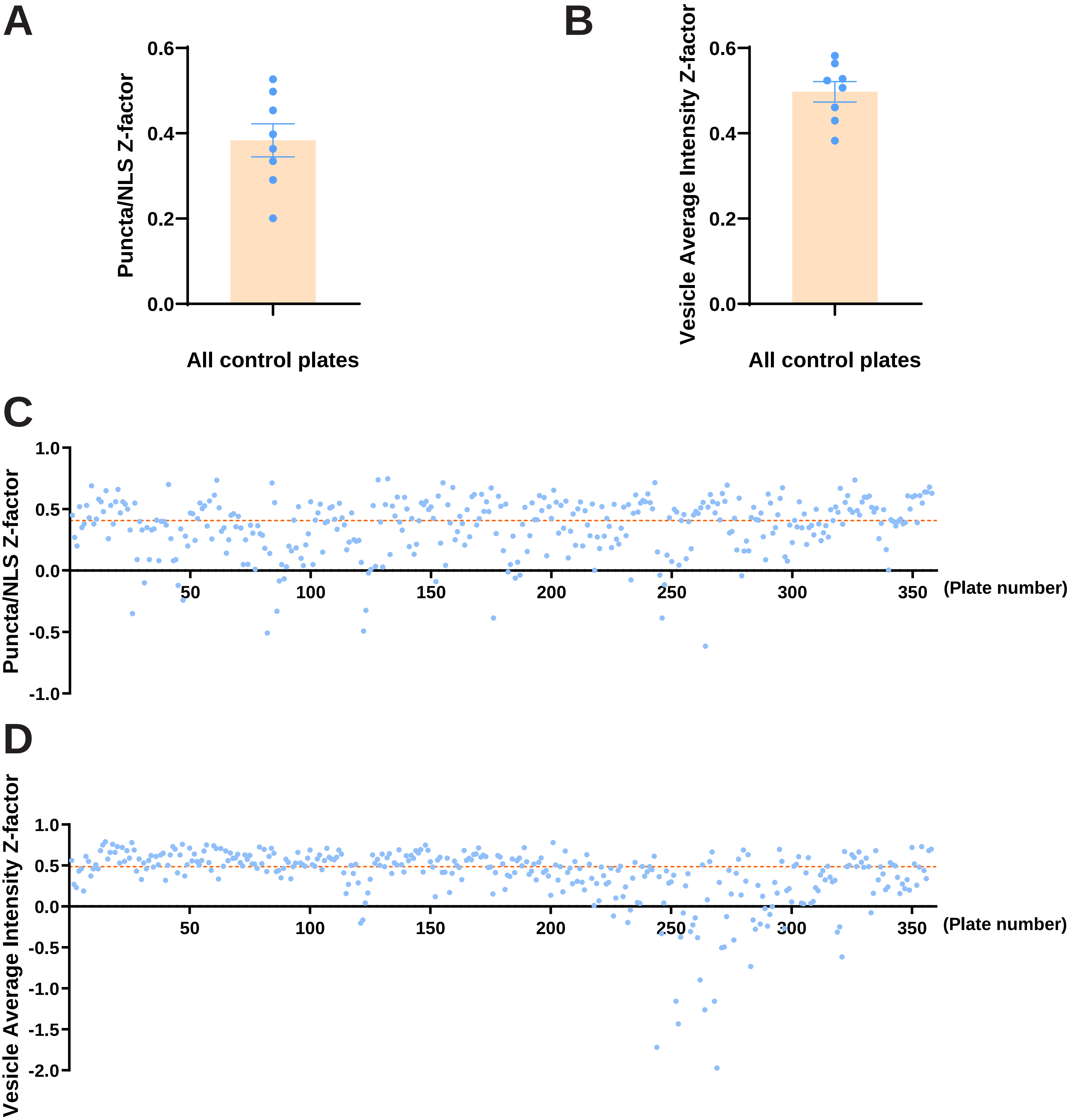

Figure S3: Documentation of robustness of scaled-up primary screening assay, related to Figure 3. ***A:*** Z-factor for Puncta/NLS readout, plotted for each of eight 96-well plates (four pairs, imaged in four consecutive runs) containing HEK293T cells transfected to express wtDLK-GFP and mCh-NLS, in which adjacent blocks of 4x3 wells were treated with DMSO vehicle of 2BP (positive control). ***B:*** As *A*, except for VAI readout. ***C:*** Z-factor for Puncta/NLS readout, plotted for each plate used within the main primary screen of 28,400 compounds. ***D:*** As *C***,** but for VAI readout. Orange dotted lines in *C* and *D* indicate the median Z-factor for all plates used in the main primary screen.

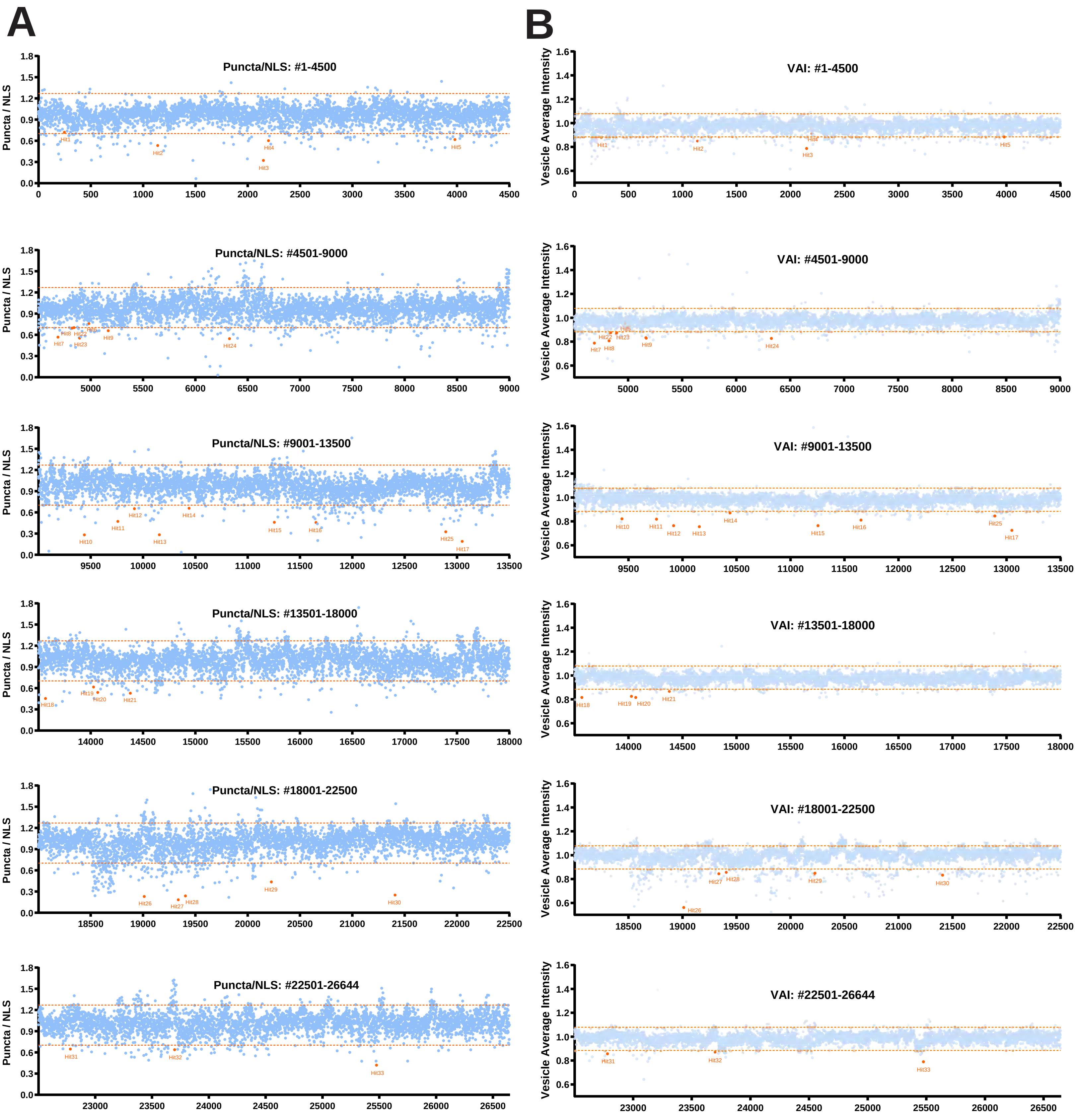

Figure S4: Expanded views (4500 compound bins) showing positions of the 33 hits identified in the primary screen on plots of (A) Puncta/NLS and (B) VAI. *Orange dots:* each hit, annotated with its corresponding number. *Blue dots:* 'Non hits'. Orange dotted lines indicate 2 standard deviations (2 SD) above and below the mean of all determinations that passed cut-offs. Note that some hit compounds identified in early rounds of screening lie within the final 2SD boundary because the 2 SD cut-off was calculated on a rolling basis as the screen proceeded. Numerical order of hits is not consecutive because compound availability and other priorities affected the order of confirmation. A single biological replicate was run for each compound in this primary screen. Data are re-plotted from Figure 3.

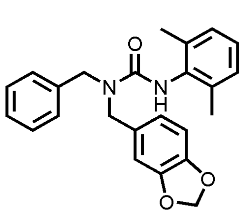

1

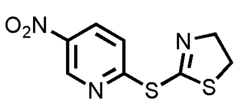

2

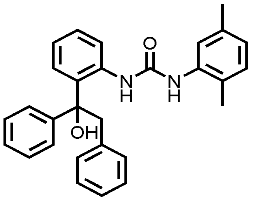

3

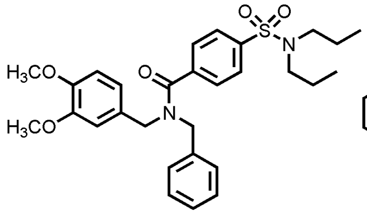

4

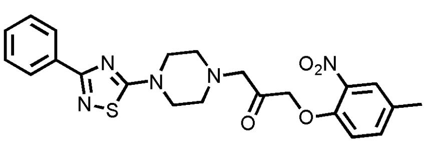

5

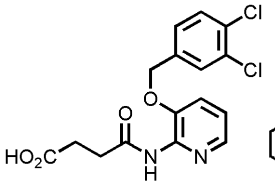

6

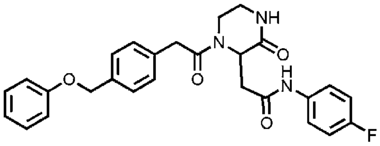

7

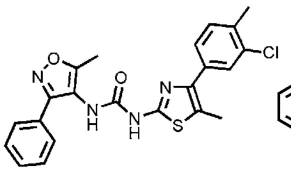

8

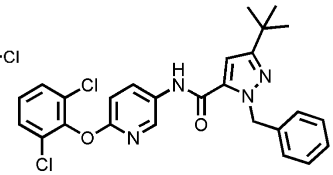

9

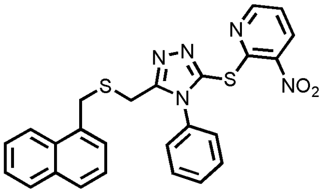

10

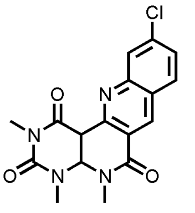

11

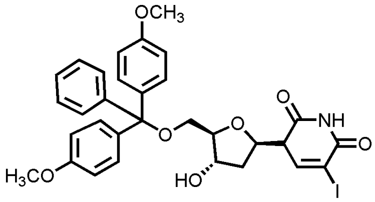

12

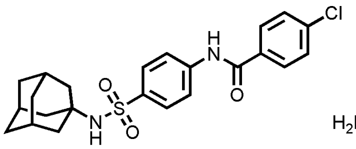

13

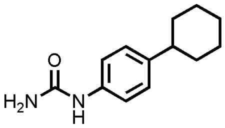

14

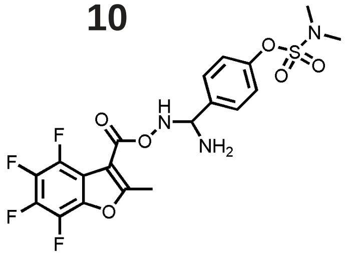

15

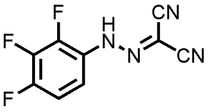

16

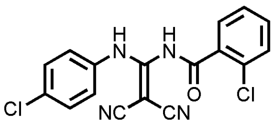

17

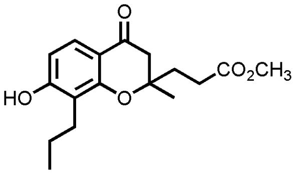

18

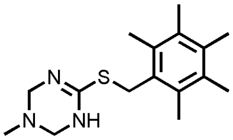

19

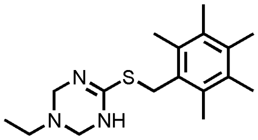

20

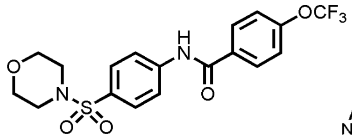

21

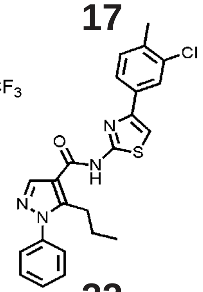

22

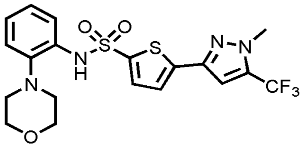

23

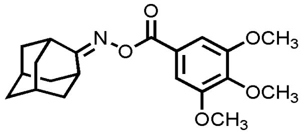

24

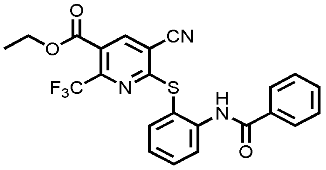

25

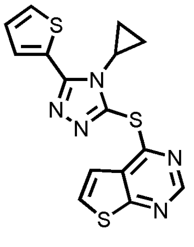

26

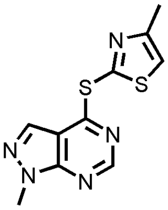

27

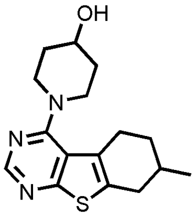

28

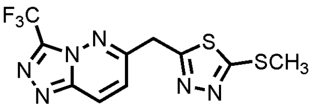

29

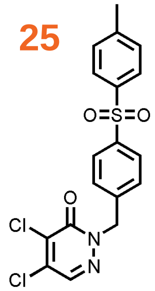

30

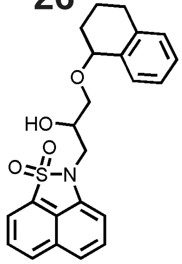

31

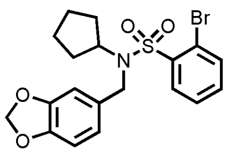

32

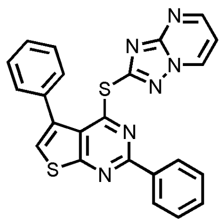

33

Figure S5: Structures of 33 compounds used in TD-induced c-Jun phosphorylation assay in Figure 4. Identifying numbers for those compounds that were followed up in neurodegeneration assay in Figure 5 are highlighted in orange.

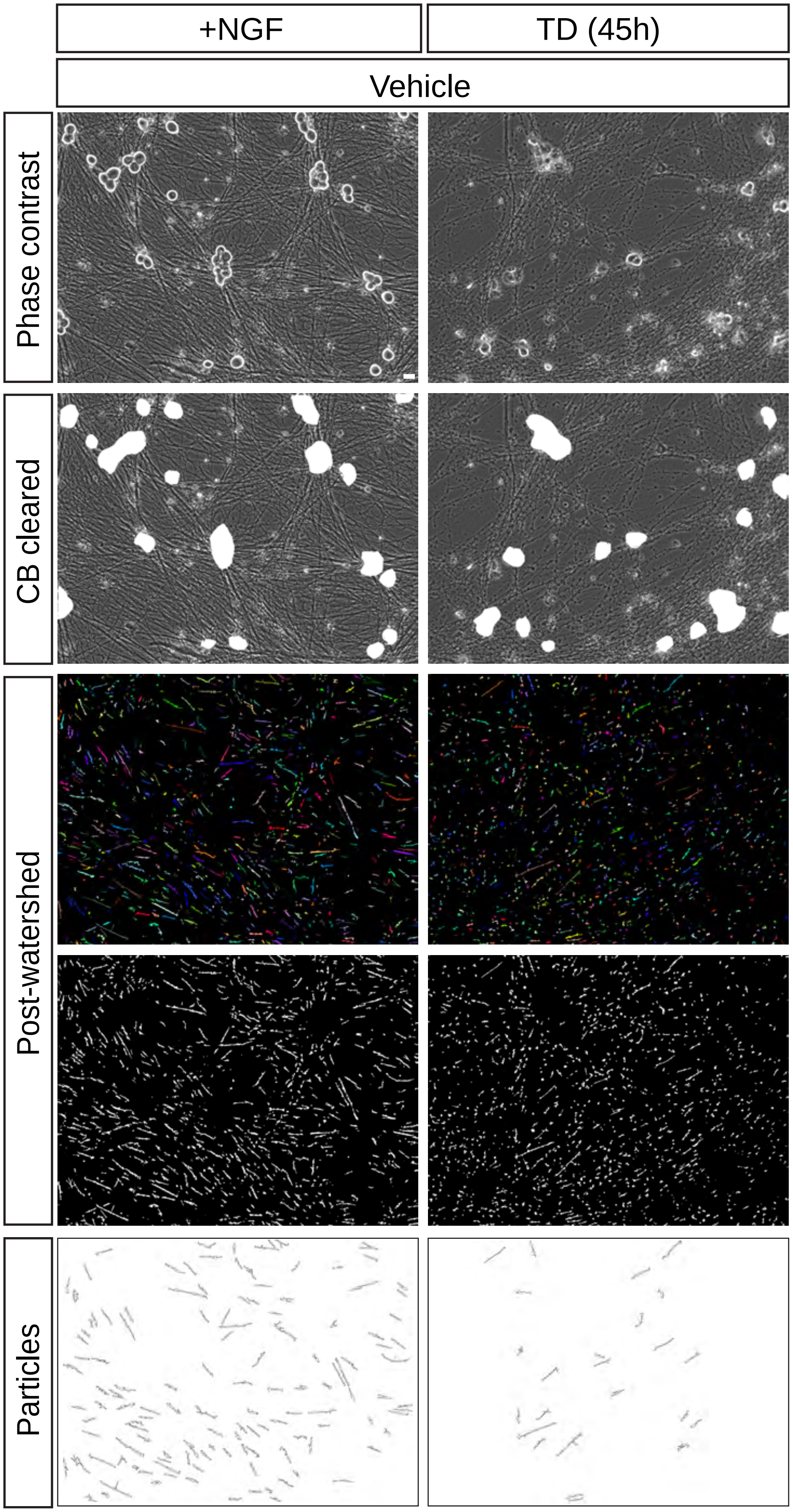

Figure S6: Examples of image processing steps used for Axon Integrity analysis in Figure 5A and 5C. *Top row:* raw phase contrast images from the indicated conditions (*left:* +NGF; *right:* TD (45h)). *2nd row:* phase contrast images after manual clearing of cell bodies. *3rd and 4th rows:* Phase contrast images after watershed segmentation to identify long unbroken axons. *Bottom row:* Particle analysis of 4th row images.Scale bar: 20µm, all panels. Representative images are shown. Similar results were obtained from 4 independent cultures

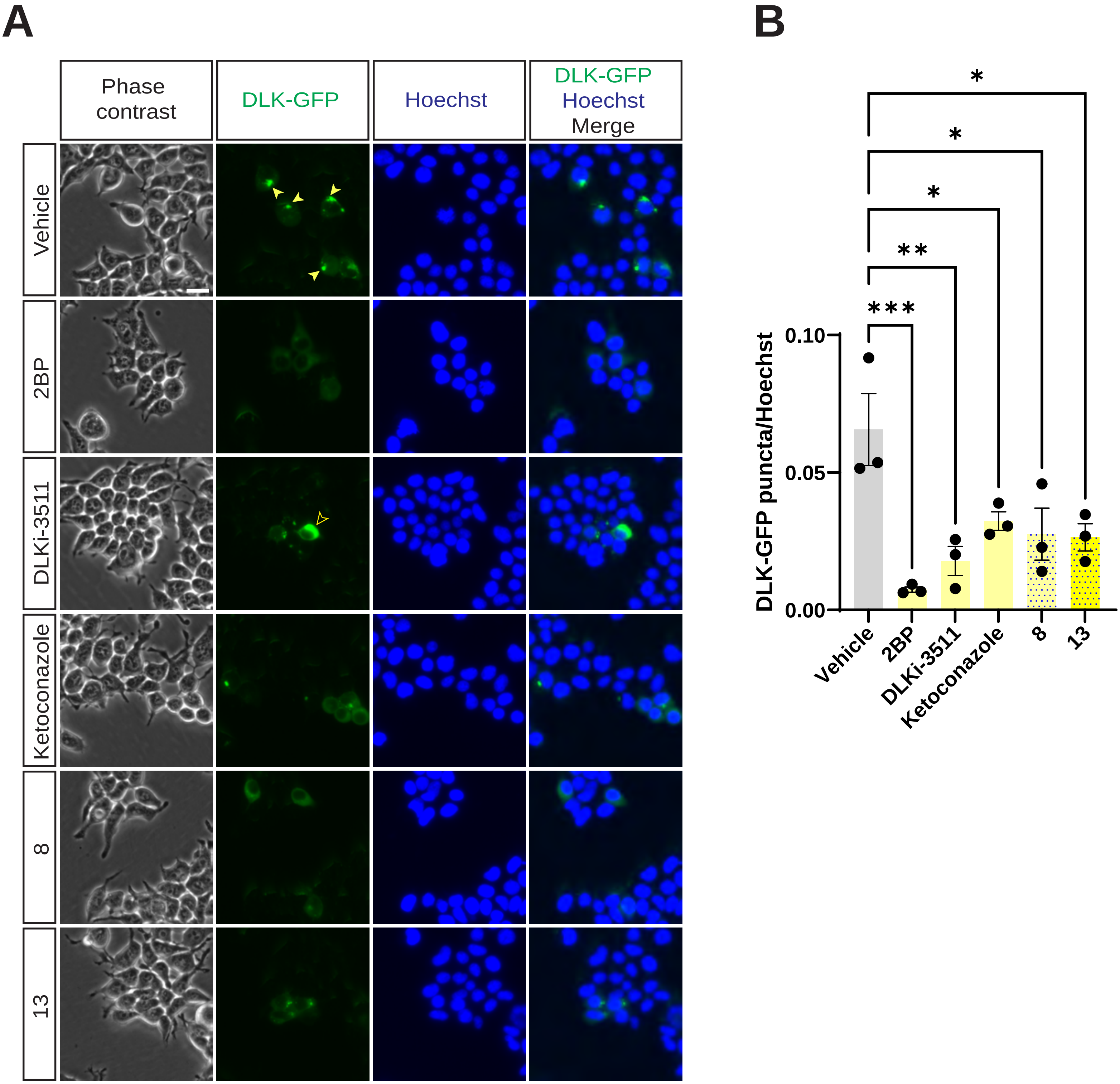

Figure S7: Confirmation of effects of key compounds in targeted follow-up of primary screening assay. *A:* Phase-contrast images (first column), DLK-GFP distribution (2nd column) and Hoechst 33342 DNA signal (3rd column) from HEK293T cells transfected to express wt DLK-GFP and then treated with the indicated compounds. The fourth column shows a merged image of the DLK-GFP and Hoechst signals. *Filled arrowheads:* DLK-GFP-positive accumulations of the size and morphology quantified as 'Puncta' in the primary screen. *Open arrowhead:* example of a large accumulation of DLK-GFP, observed in a subset of GNE-3511-treated cells. ***B:*** Quantified data for multiple determinations per condition from *A.* Cells treated with 2BP, ketoconazole (most-potent hit from pilot screen), **8** or **13** reduce DLK-GFP puncta. Large accumulations of DLK-GFP in some GNE-3511-treated cells are larger than the size cut-off for defined 'puncta', so cells treated with this compound also show a reduced number of DLK-GFP 'puncta'. Statistical significance versus vehicle was as follows: 2BP: ***; p=0.0005; DLKi-3511: **; p = 0.0026; Ketoconazole: *; p=0.0295; **8**: *; p=0.0132; **13**: *; p=0.0108; ANOVA, Dunnett's post hoc test.Scale bar: 20μm, all panels. Representative images are shown. Similar results were obtained from 3 independent sets of transfected cells.

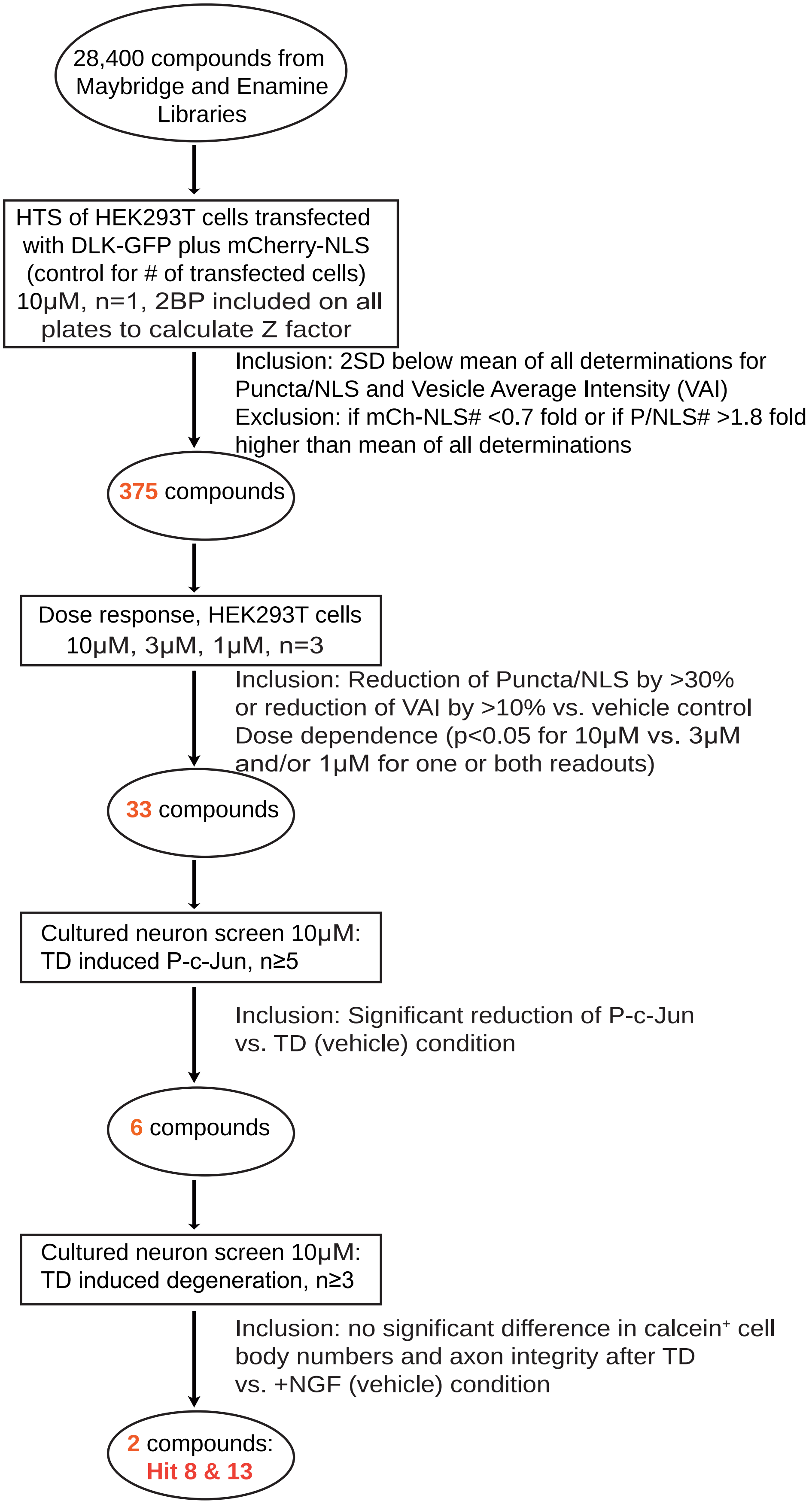

Figure S8: Specific steps and inclusion/exclusion criteria for primary and secondary screening assays. See also Table S2.

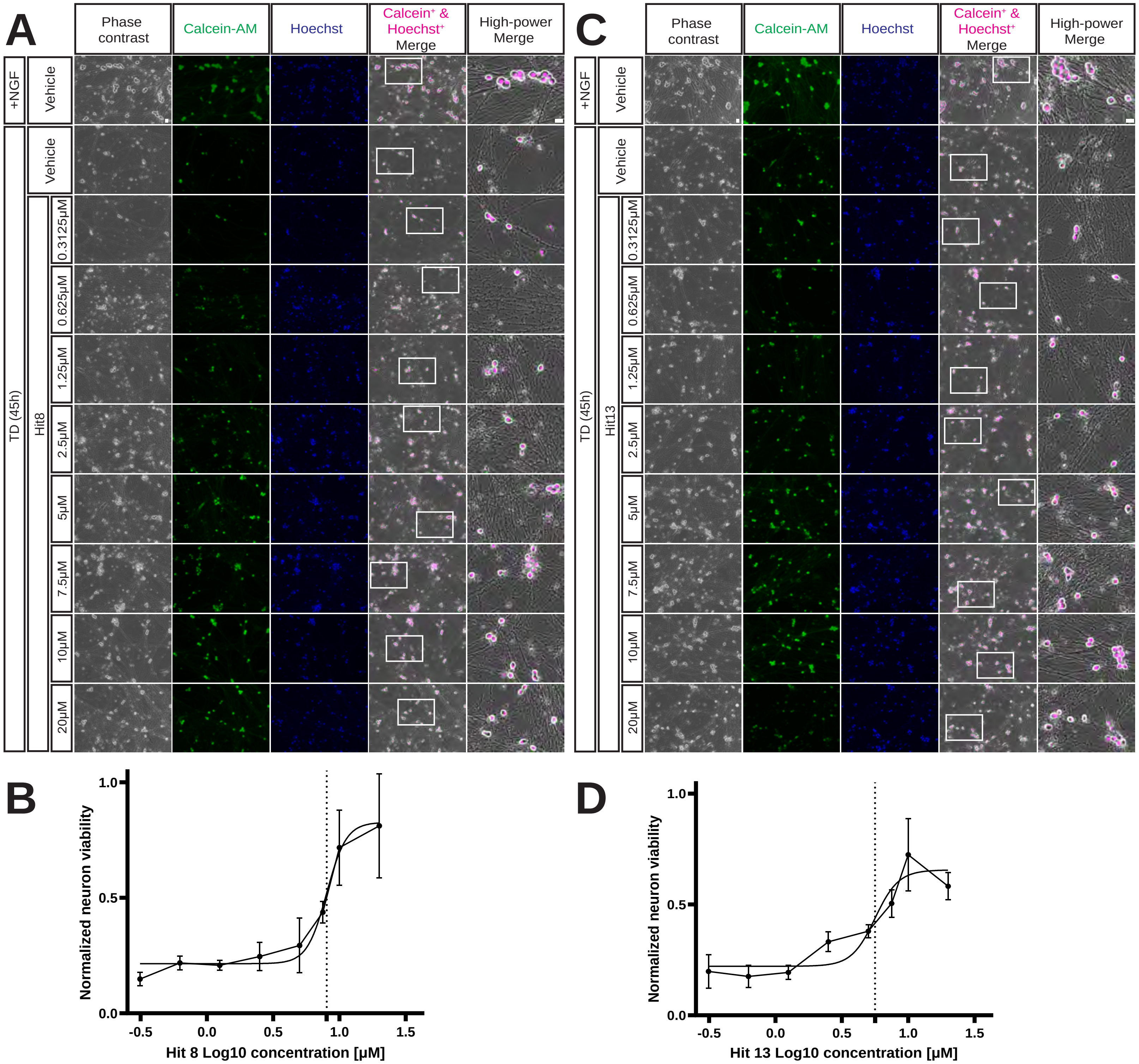

Figure S9: IC50 determination for hit compounds in cell body viability assay. ***A:*** Phase contrast images (1st column), Calcein-AM signal (2nd column) and Hoechst 33342 DNA signal (3rd column) of cultured DRG neurons that were maintained in NGF (+NGF) or subjected to TD for 45h in the presence of vehicle (DMSO) or the indicated concentrations of **8**. *Fourth column:* overlay of the phase contrast image with the Calcein-AM/Hoechst double-positive signal (latter false-colored in magenta). *Fifth column:* magnified views of the boxed region in the corresponding fourth column. Scale bars, 20 μm (all panels). ***B:*** Number of Calcein-AM/Hoechst double-positive cells per field (a measure of viable cells), plotted against $\log_{10}$ concentration of **8**, quantified from images from *A*. ***C, D:*** As *A, B,* but for indicated concentrations of **13**. The IC50 value (X-intercept, dotted line in *B* and *D*) for **8** is 8.0μM, and for **13** is 5.6 μM determined from a nonlinear curve fit of the data as described in Methods. Scale bar: 20μm, all panels.

In *A* and *B*, representative images are shown from the following numbers of independent cultures: +NGF: 7; TD/DMSO: 7; TD/03125μM **8**: 3; TD/0.625μM **8**: 3; TD/1.25μM **8**: 3; TD/2.5μM **8**: 3; TD/5μM **8**: 4; TD/7.5μM **8**: 4; TD/10μM **8**: 4; TD/20μM **8**: 3. In *C* and *D*, representative images are shown from the following numbers of independent cultures: +NGF: 7; TD/DMSO: 6; TD/0.3125μM **13:** 3; TD/0.625μM **13**:3; TD/1.25μM **13**: 3; TD/2.5μM **13**: 3; TD/5μM **13**: 3; TD/7.5μM **13**: 3; TD/10μM **13**: 5; TD/20μM **13**: 4.

Data in *B* and *D* are plotted as Mean +/- SD for concentrations of **8** and **13** from 0.3125-20μM, respectively.

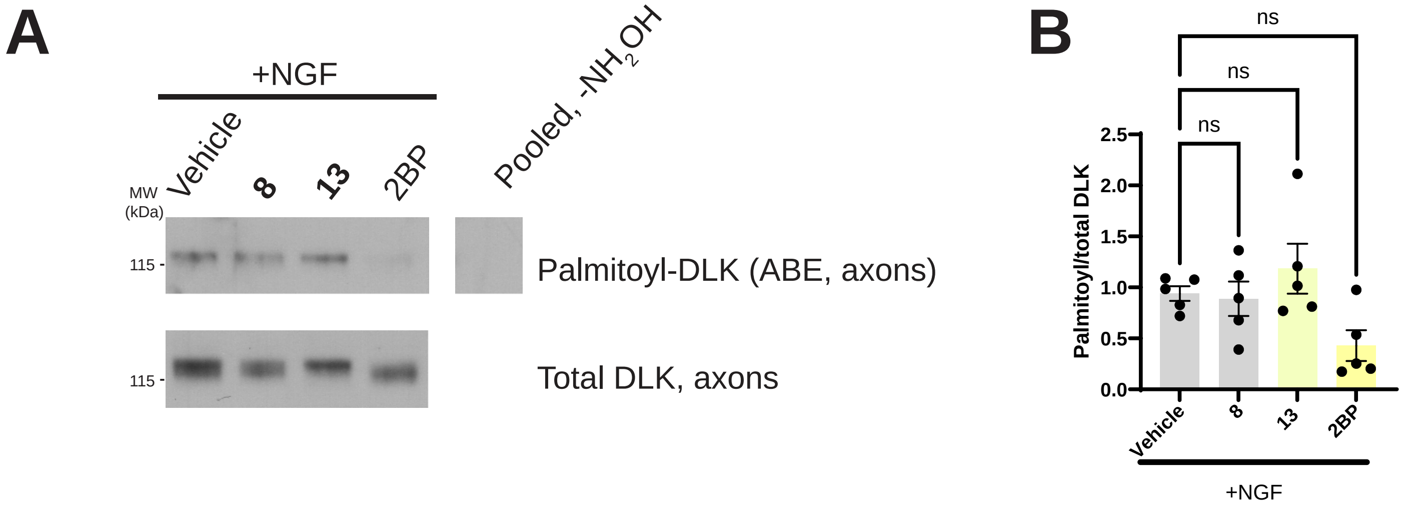

Figure S10: No effect of 8 or 13 on DLK palmitoylation in the presence of NGF. ***A:*** Western blots of ABE (palmitoyl-) fractions of DRG axonal lysates that had been treated as indicated for 3h in the continued presence of NGF prior to lysis. The righthand lane shows a side-by-side exposure from a parallel control sample omitting the key ABE reagent NH_2OH, run on the same gel, with intervening spacer lanes cropped. ***B:*** Quantified data from *A* confirm that neither **8** nor **13** reduces the palmitoylation of DLK below that seen in vehicle-treated cells. Axonal palmitoylation of DLK in neurons treated with 2BP trends towards a decrease but does not reach significance. Ns: not significant, ANOVA, Dunnett's post hoc test. 5 independent cultures per condition.

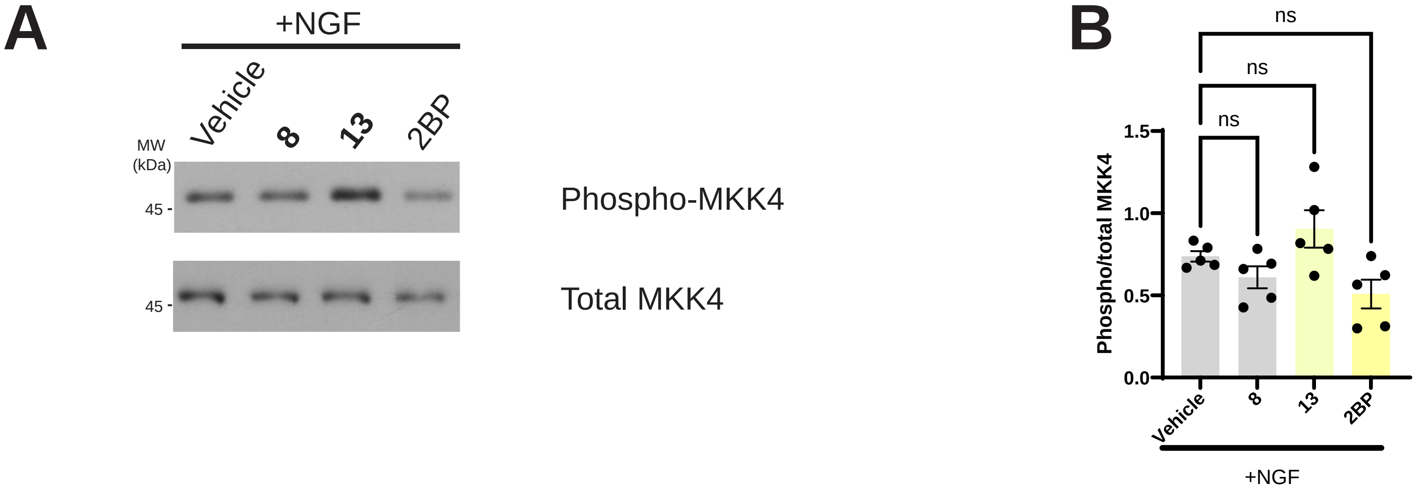

Figure S11: No effect of 8 or 13 on MKK4 phosphorylation in the presence of NGF. ***A:*** Input lysates from Fig S10 were blotted to detect phospho- and total MKK4. ***B:*** Quantified data from *A* confirm that neither **8** nor **13** reduces the MKK4 phosphorylation below that seen in vehicle-treated cells. Ns: not significant, ANOVA, Dunnett's post hoc test. 5 independent cultures per condition.

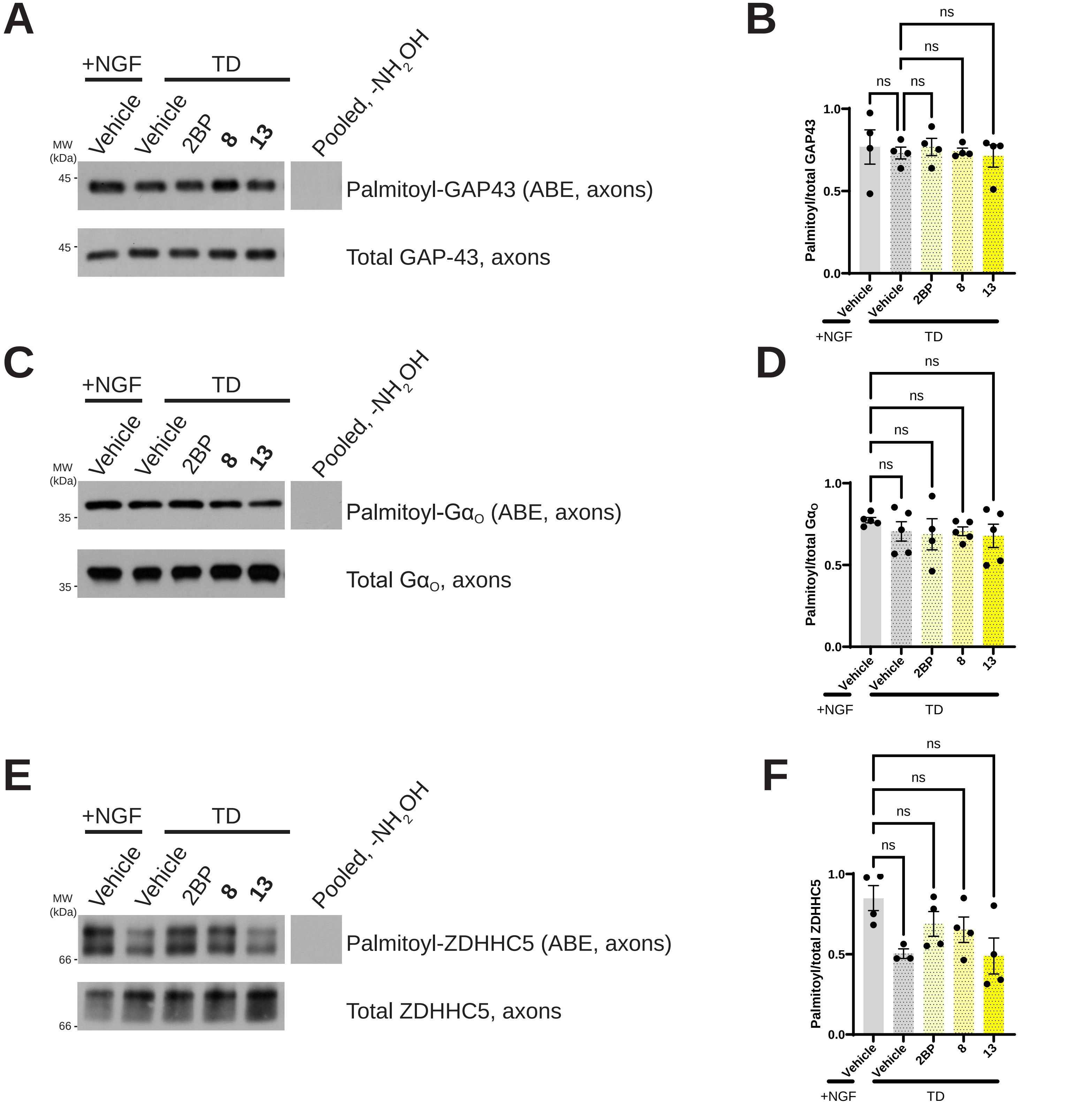

Figure S12: Hit compounds do not reduce palmitoylation of other axonal proteins tested. ***A:*** Axonal samples from experiments in Figure 6C were immunoblotted to detect the abundant axonal palmitoyl-protein GAP-43. ***B:*** Quantified data from multiple determinations from *A* confirm that GAP-43 palmitoylation is unaltered by TD in the presence or absence of 2BP, **8** or **13**. Ns: not significant, ANOVA, Dunnett's post hoc test. ***C:*** As *A*, except that samples were blotted to detect $G\alpha_o$, a G protein alpha subunit highly expressed in DRG neurons. ***D:*** Quantified data from multiple determinations from *A* confirm that $G\alpha_o$ palmitoylation is unaltered by TD in the presence or absence of 2BP, **8** or **13**. Ns: not significant. ANOVA, Dunnett's post hoc test. ***E:*** As *A*, except that samples were blotted to detect ZDHHC5, a PAT that is abundant in DRG axons. The righthand lanes on panels *A, C* and *E* each show a side-by-side exposure from a parallel control sample omitting the key ABE reagent NH_2OH, run on the same gel, with intervening spacer lanes cropped. ***F:*** Quantified data from multiple determinations from *A* confirm that ZDHHC5 palmitoylation is unaltered by TD in the presence or absence of 2BP, **8** or **13**. Ns: not significant. ANOVA, Dunnett's post hoc test. GAP-43: data from 4 independent cultures. $G\alpha_o$, data from 5 independent cultures except 2BP condition (4 independent cultures). ZDHHC5: data from 4 independent cultures except TD/vehicle condition (3 independent cultures)

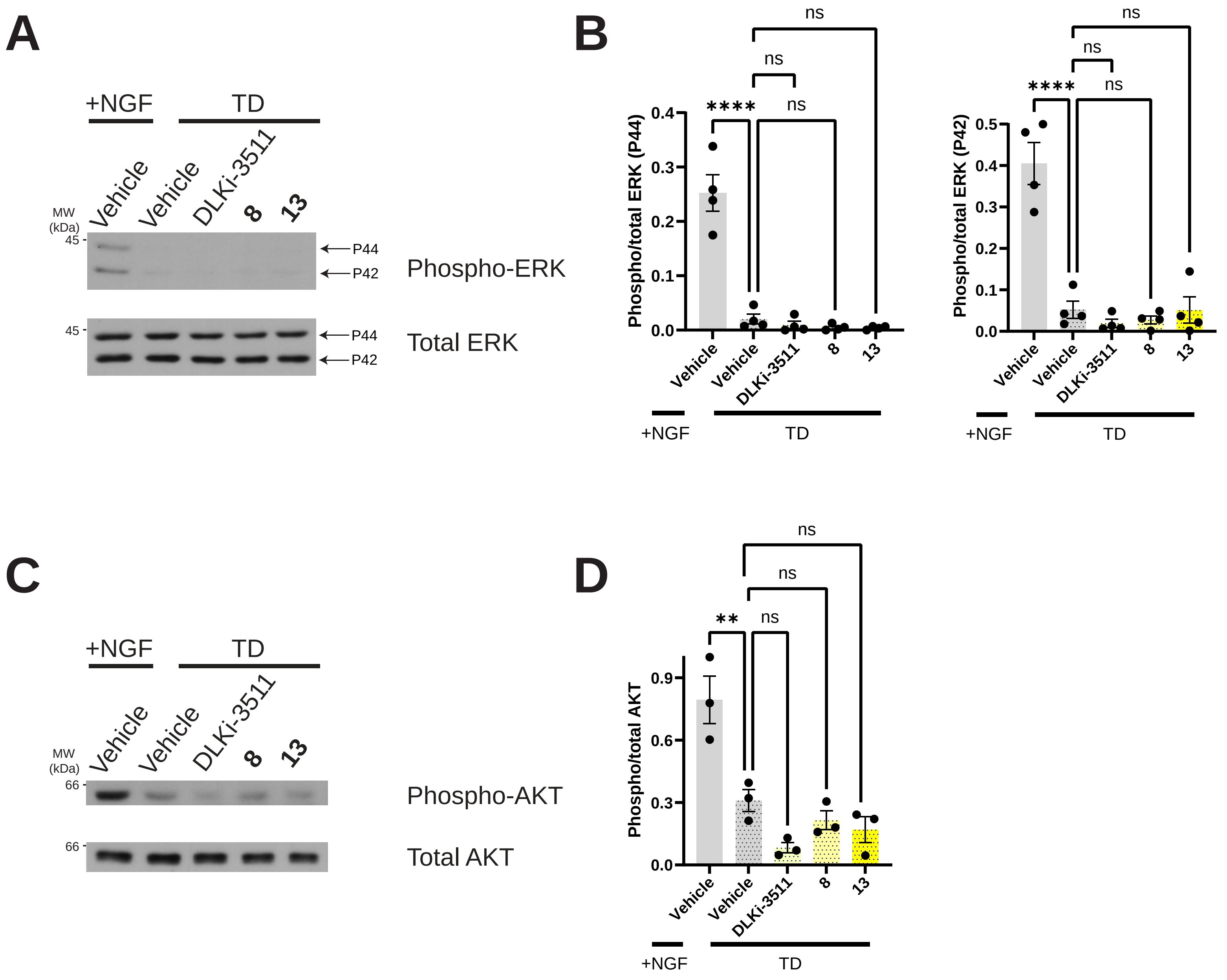

Figure S13: Novel compounds do not prevent TD-induced shutdown of ERK and Akt signaling pathways. ***A:*** Lysates from experiments in Fig 6E were immunoblotted to detect phosphorylated and total forms of ERK (p42, p44, indicated with arrows). ***B:*** Phospho:total ratios for p44 (upper) and p42 (lower) forms of ERK, quantified from multiple determinations from *A.* *****;* p <0.0001, ANOVA, Dunnett's post hoc test. 4 independent cultures per condition. ***C:*** As *A*, except that lysates were blotted to detect Akt phosphorylated at T308 and total Akt. ***D:*** Phospho:total ratios for Akt, quantified from multiple determinations from *A.* **; p =0.0034, ANOVA, Dunnett's post hoc test. Data from 3 independent cultures.

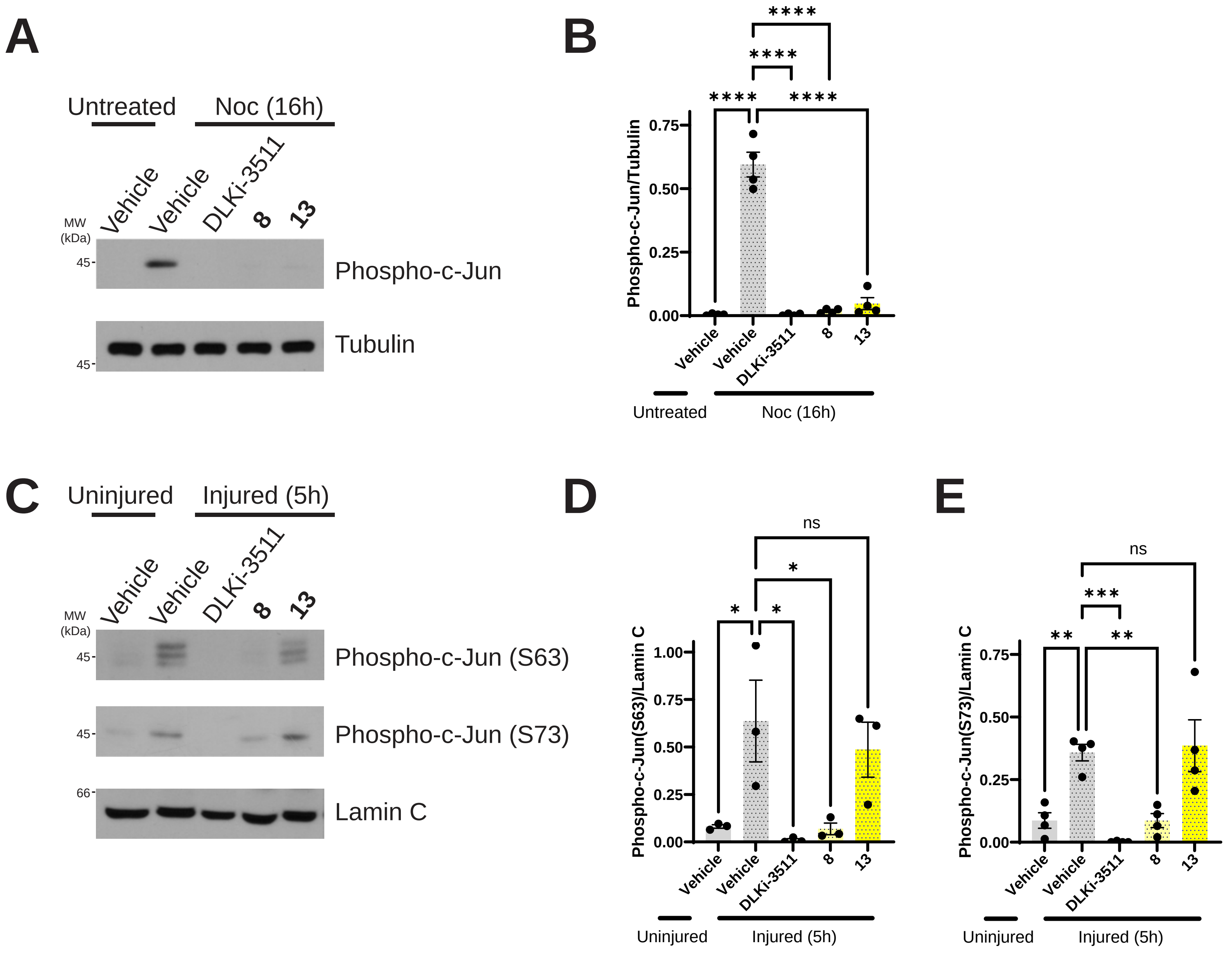

Figure S14: Novel compounds differentially affect other forms of DLK-dependent signaling. ***A:*** DRG neurons were pre-treated for 15 min (as in [35]) with the indicated compounds or with vehicle and then subjected to 16h treatment with low dose nocodazole (Noc) or were left untreated. Lysates were blotted with the indicated antibodies ***B:*** Quantified p-c-Jun:tubulin, for multiple determinations from *A*. Both 8 and 13 inhibit nocodazole-induced c-Jun phosphorylation. ****; p<0.0001, ANOVA with Dunnett's post hoc test. 4 independent cultures per condition. ***C:*** DRG neurons in spot cultures were treated with the indicated compounds or with vehicle and then immediately subjected to distal axotomy or were left uninjured. Lysates were blotted with anti-phospho c-Jun S63 antibody (used in other experiments in this study e.g. panel *A* above). Because anti phospho cJun (S63) antibody detects a ladder of bands in lysates from axotomized samples, c-Jun phosphorylation at a nearby S73 site was also assessed. ***D:*** Quantified data from C for anti-phospho c-Jun S63, relative to lamin C. **8** inhibits axotomy-induced c-Jun phosphorylation at S63 but **13** does not. Uninjured (vehicle) vs. Injured (vehicle): p=0.0077; Injured (vehicle) vs. Injured (DLKi-3511): p=0.0008; Injured (vehicle) vs. Injured (**8**): p=0.0076; Injured (vehicle) vs.Injured (**13**): ns. ANOVA with Dunnett's post hoc test. 3 independent cultures per condition. ***E:*** As *D*, but for anti-p-c-Jun(S73):lamin C, for multiple determinations from *C.* **8** inhibits axotomy-induced c-Jun phosphorylation at S73 but 13 does not. Uninjured (vehicle) vs. Injured (vehicle): p=0.0235; Injured (vehicle) vs. Injured (DLKi-3511): p=0.0116; Injured (vehicle) vs. Injured (**8**): p=0.0208; Injured (vehicle) vs. Injured (**13**): ns. ANOVA with Dunnett's post hoc test. 4 independent cultures per condition.

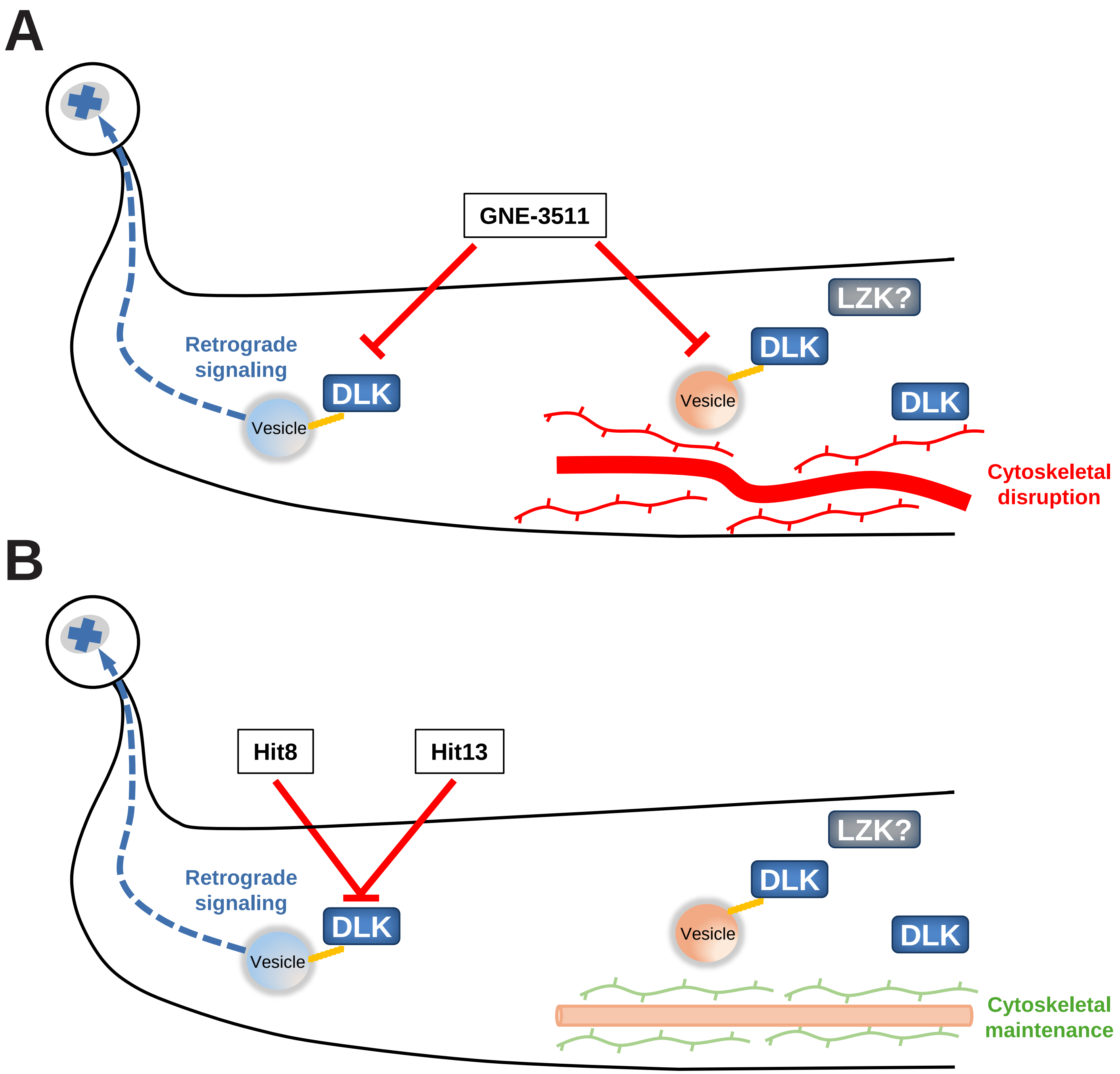

Figure S15: Model summarizing the major findings of this study. ***A:*** Compounds such as GNE-3511 that inhibit DLK's kinase domain block all cellular pools of DLK. Such compounds prevent DLK-dependent retrograde signaling but also cause disruption of the axonal cytoskeleton and vesicle-based transport. ***B:*** Novel compounds (Hit 8 and 13) selectively inhibit stimulus-dependent palmitoylation of DLK, blocking retrograde signaling while leaving the axonal cytoskeleton intact. Retrograde signaling blocked by GNE-3511, **8** and **13** is likely due to action of these compounds on DLK signaling, because similar block is seen with DLK knockout and knockdown. However, GNE-3511 and related compounds also inhibit DLK's close paralog LZK, and potentially other kinases[39, 54]. A key future question is the extent to which effects of DLK kinase inhibitors on the cytoskeleton and/or other effects reported in the clinical trial of one such inhibitor, are due to on-target action, compared to these potential off-targets.
