# Supplementary material for: Inhibiting acute, axonal DLK palmitoylation is neuroprotective and avoids deleterious effects of cell-wide DLK inhibition
Source: Nat Commun. 2025 Apr 3;16:3031. doi: 10.1038/s41467-025-58036-6 (PMC11968826; doi:10.1038/s41467-025-58036-6)
Supplement: Supplementary file 2 — Reporting Summary [file 41467_2025_58036_MOESM2_ESM.pdf]

nature portfolio

Corresponding author(s): Gareth M Thomas

Last updated by author(s): Feb 21, 2025

Reporting Summary

Nature Portfolio wishes to improve the reproducibility of the work that we publish. This form provides structure for consistency and transparency in reporting. For further information on Nature Portfolio policies, see our Editorial Policies and the Editorial Policy Checklist.

Statistics

For all statistical analyses, confirm that the following items are present in the figure legend, table legend, main text, or Methods section.

n/a	Confirmed	
☐	☒	The exact sample size (*n*) for each experimental group/condition, given as a discrete number and unit of measurement
☐	☒	A statement on whether measurements were taken from distinct samples or whether the same sample was measured repeatedly
☐	☒	The statistical test(s) used AND whether they are one- or two-sided<br>*Only common tests should be described solely by name; describe more complex techniques in the Methods section.*
☐	☒	A description of all covariates tested
☐	☒	A description of any assumptions or corrections, such as tests of normality and adjustment for multiple comparisons
☐	☒	A full description of the statistical parameters including central tendency (e.g. means) or other basic estimates (e.g. regression coefficient) AND variation (e.g. standard deviation) or associated estimates of uncertainty (e.g. confidence intervals)
☐	☒	For null hypothesis testing, the test statistic (e.g. *F*, *t*, *r*) with confidence intervals, effect sizes, degrees of freedom and *P* value noted<br>*Give P values as exact values whenever suitable.*
☒	☐	For Bayesian analysis, information on the choice of priors and Markov chain Monte Carlo settings
☒	☐	For hierarchical and complex designs, identification of the appropriate level for tests and full reporting of outcomes
☒	☐	Estimates of effect sizes (e.g. Cohen's *d*, Pearson's *r*), indicating how they were calculated

Our web collection on statistics for biologists contains articles on many of the points above.

Software and code

Policy information about availability of computer code

Data collection	Images in Figure 1 and Figure 7 were acquired using Nikon NIS Elements software<br>Images in Figure 2A, Figure 6 and Figure 8 were acquired using Leica LASX (version 3.7.4.23463; Leica Inc.).<br>Images in Figure 2E were acquired using Olympus Cellsens 4.2 software (Evident)<br>Images in Fig 5 were acquired using Evos M5000 software (Invitrogen)
Data analysis	Image processing and quantification was performed using Fiji / ImageJ (Win64, Version 1.53t). Statistical analysis and plotting of graphs was performed using GraphPad Prism 10.2.2

For manuscripts utilizing custom algorithms or software that are central to the research but not yet described in published literature, software must be made available to editors and reviewers. We strongly encourage code deposition in a community repository (e.g. GitHub). See the Nature Portfolio guidelines for submitting code & software for further information.

Data

Policy information about availability of data

All manuscripts must include a data availability statement. This statement should provide the following information, where applicable:

- Accession codes, unique identifiers, or web links for publicly available datasets
- A description of any restrictions on data availability
- For clinical datasets or third party data, please ensure that the statement adheres to our policy

The minimal dataset necessary to interpret, verify and extend the research in this study is provided in the Supplementary Information and Source Data files.

Research involving human participants, their data, or biological material

Policy information about studies with human participants or human data. See also policy information about sex, gender (identity/presentation), and sexual orientation and race, ethnicity and racism.

Reporting on sex and gender	N/A
Reporting on race, ethnicity, or other socially relevant groupings	N/A
Population characteristics	N/A
Recruitment	N/A
Ethics oversight	N/A

Note that full information on the approval of the study protocol must also be provided in the manuscript.

Field-specific reporting

Please select the one below that is the best fit for your research. If you are not sure, read the appropriate sections before making your selection.

☒ Life sciences ☐ Behavioural & social sciences ☐ Ecological, evolutionary & environmental sciences

For a reference copy of the document with all sections, see nature.com/documents/nr-reporting-summary-flat.pdf

Life sciences study design

All studies must disclose on these points even when the disclosure is negative.

Sample size	Sample sizes are indicated in the legend of each Figure and Supplementary Figure. No statistical methods were used to predetermine sample size. Estimates were based on our prior experience and/or reference to similar methods in the literature.
Data exclusions	We did not exclude any data from the reported experiments
Replication	Experimental findings were reliably reproduced. The number of animals or cultures used for a given experiment is indicated in the corresponding Figure legend and individual data points are plotted for each graph to show the spread of data.
Randomization	Wild type mice were randomly assigned to each experimental condition. Plates of cultured neurons were also randomly assigned. For high content screening in 96-well plates, the first and last column of each plate were used for vehicle control conditions (4 wells per condition, duplicated) and 2-bromopalmitate (positive control tool compound, 4 wells per condition, duplicated). Other compounds were randomly assigned across wells.
Blinding	For acquisition of fluorescent signals in cultured, uninfected neurons (Figure 1, Figure 7) a field of view was first selected based on NF200 signal (which is most constant between treatment conditions). Images in other channels were acquired from the same field of view, without the experimenter having prior knowledge of that field. Additional images from the same coverslip were acquired from directly adjacent but non-overlapping fields. Blinding was not used for other experiments.

Reporting for specific materials, systems and methods

We require information from authors about some types of materials, experimental systems and methods used in many studies. Here, indicate whether each material, system or method listed is relevant to your study. If you are not sure if a list item applies to your research, read the appropriate section before selecting a response.

Materials & experimental systems

n/a	Involved in the study	
	x	Antibodies
	x	Eukaryotic cell lines
x		Palaeontology and archaeology
	x	Animals and other organisms
x		Clinical data
x		Dual use research of concern
x		Plants

Methods

n/a	Involved in the study	
x		ChIP-seq
x		Flow cytometry
x		MRI-based neuroimaging

Antibodies

Antibodies used

The following primary antibodies, raised in the indicated species, were purchased from Cell Signaling Technology: phospho–c-Jun (Ser63) (rabbit, #91952, used at 1:500 dilution for western blot (WB), 1:100 for immunocytochemistry and immunohistochemistry (ICC, IHC); phospho–c-Jun (Ser73) (rabbit, #3270, used at 1:500 for WB); myc (rabbit, #2278 used at 1:100 for ICC); alpha-tubulin (mouse, #3873, used at 1:2000 for WB); phospho-MKK4 (rabbit, #4514, used at 1:1000 for WB); pan-MKK4 (rabbit, #9152 used at 1:1000 for WB); Lamin A/C (mouse, #4777 used at 1:400 for WB), phospho-Akt (Thr308) rabbit #9275, used at 1:250 for WB), Akt (rabbit, #4691, used at 1:1000 for WB), pan-ERK (rabbit, #4635, used at 1:1000 for western blot), phospho-ERK1/2 (mouse #9106, used at 1:250 for WB),. Additional antibodies were from the following indicated suppliers: anti-NGF (sheep, CedarLane, #CLMCNET-031, used at 1:40 dilution for live cell assays); DLK/MAP3K12 (rabbit, Genetex, #GTX124127, used at 1:5000 for WB); β3 tubulin (mouse, BioLegend, TUJ1, #MMS-435P, used at 1:1000 for ICC), NF-200 (mouse, MilliporeSigma #NO-142, used at 1:1000 for ICC), VAMP2 (mouse, Synaptic Systems, #104211SY, used at 1:1000 for ICC), Brn3a (mouse, Millipore Sigma, # MAB1585, used at 1:100 for IHC), GAP-43 (Go)   (rabbit, ProteinTech 12-635-1-AP, used at 1:5000 for WB); ZDHHC5 (rabbit, MilliporeSigma HPA014670, used at 1:2000 for WB).

Validation

anti-NGF was validated functionally by ability to induce degeneration and induce changes in axonal transport. Manufacturer states that antibody is validated by: " immunosympathectomy of newborn mice and rats
- neutralization of NGF activity in tissue culture and in vivo; two-site ELISA and Western blot of NGF. On Western blots, the I-125labelled IgG fraction of the anti-NGF labels only the 13.5 kDa bands corresponding
to the 2.5S NGF monomer. No antibodies bind to mouse serum proteins, renin or epidermal growth factor."

Phospho-c-jun antibodies were verified by detection of single band of predicted molecular weight, appearance blocked by inhibition of c-Jun N-terminal kinase or DLK (known results from our own laboratory and from the literature). Manufacturer confirms phospho-specificity by loss of signal following lambda and calf intestinal phosphatase treatment.

DLK/MAP3K12 antibody was validated by loss of western blot and immunofluorescent signal in cultures virally infected to express DLK shRNA

β3 tubulin antibody was validated by the manufacturer and in-house to detect only a single band of predicted molecular weight. Used in >750 publications.

myc (rabbit monoclonal) antibody was validated by western blot and immunofluorescent assays - only detects signal in cultures transfected or infected to express myc-tagged proteins. Similarly validated by manufacturer.

alpha-tubulin antibody was validated by the manufacturer and in-house to detect only a single band of predicted molecular weight. Used in >900 publications.

Phospho-MKK4 antibody was validated by the manufacturer and in-house to detect only a single band of predicted molecular weight. Signal increases in samples from cells in which DLK/JNK pathway is known to be activated. Signal blocked by inhibition of DLK (direct upstream kinase). These are known results from our own laboratory and from the literature.

Pan-MKK4 antibody was validated by the manufacturer and in-house to detect only a single band of predicted molecular weight. Used in >100 publications.

GAP-43 antibody was validated by the manufacturer and in-house to detect only a single band of predicted molecular weight. . This antibody has been used in >100 publications.

ZDHHC5 antibody detects a single band of predicted molecular weight. We have previously confirmed that a specific shRNA against ZDHHC5 eliminates this band.

G alpha o antibody was validated by the manufacturer and in-house to detect only a single band of predicted molecular weight. Used in 10 publications

Eukaryotic cell lines

Policy information about cell lines and Sex and Gender in Research

Cell line source(s)	HEK293T cells were from ATCC
Authentication	Cell line was authenticated by ATCC using STR profiling and was found to be an exact match to CRL-3216 (HEK293T) in the ATCC STR database.
Mycoplasma contamination	The cells were tested for mycoplasma and were negative.
Commonly misidentified lines (See ICLAC register)	N/A

Animals and other research organisms

Policy information about studies involving animals; ARRIVE guidelines recommended for reporting animal research, and Sex and Gender in Research

Laboratory animals	C57Bl/6 mice were used for experiments at 5-8 weeks of age. Similar numbers of male and female mice were used but data were not segregated by sex. For cultured neuron experiments from embryonic rats, timed pregnant Sprague Dawley rats were obtained from Charles River. At 16 days of gestation, embryos of both sexes were dissected without genotyping and all material was pooled.
Wild animals	N/A
Reporting on sex	Cultured rat neurons were obtained from embryos of both sexes and material was pooled. For experiments involving mice, 14 male and 13 female mice, assigned randomly across conditions) were obtained from Jackson Laboratories and used for this study. Data disaggregated by sex are provided in the Source Data file. Data in the main mansucript are not disaggregated due to low overall n number, as recommended by Nature Communications policy.
Field-collected samples	N/A
Ethics oversight	The IACUC of Temple University approved all studies involving animals.

Note that full information on the approval of the study protocol must also be provided in the manuscript.

Plants

Seed stocks	*Report on the source of all seed stocks or other plant material used. If applicable, state the seed stock centre and catalogue number. If plant specimens were collected from the field, describe the collection location, date and sampling procedures.*
Novel plant genotypes	*Describe the methods by which all novel plant genotypes were produced. This includes those generated by transgenic approaches, gene editing, chemical/radiation-based mutagenesis and hybridization. For transgenic lines, describe the transformation method, the number of independent lines analyzed and the generation upon which experiments were performed. For gene-edited lines, describe the editor used, the endogenous sequence targeted for editing, the targeting guide RNA sequence (if applicable) and how the editor was applied.*
Authentication	*Describe any authentication procedures for each seed stock used or novel genotype generated. Describe any experiments used to assess the effect of a mutation and, where applicable, how potential secondary effects (e.g. second site T-DNA insertions, mosiacism, off-target gene editing) were examined.*
